# **Diagnostic value of CT scan findings for anastomotic leakage after low anterior resection without ileostomy in rectal cancer**

**DOI:** 10.1038/s41598-025-06712-4

**Published:** 2025-07-01

**Authors:** Aidin Yaghoobi Notash, Ehsan Sadeghian, Zahra Moghimi, Yasaman Rahimi, Ehsan Sobhanian

**Affiliations:** 1 General Surgeon and Faculty Member, Tehran University of Medical Sciences, Tehran, Iran; 2 Department of Surgery, Shariati Hospital, Tehran University of Medical Sciences, Tehran, Iran; 3 Department of Gynecology, Yas Hospital, Tehran University of Medical Sciences, Tehran, Iran; 4 School of medicine, Shahid Beheshti University of Medical Science, Tehran, Iran; 5 Department of Surgery, Sina Hospital, Tehran University of Medical Sciences, Tehran, Iran

**Keywords:** Abscess, Anastomotic leakage, Laparoscopy, Neoadjuvant chemotherapy, CT scan, Rectal Cancer, Rectosigmoidoscopy, Recurrence., Gastroenterology, Medical research, Oncology

## Abstract

Anastomotic leakage (AL) is one of the most serious complications following colorectal surgery, particularly in patients who have undergone low anterior resection (LAR) for rectal cancer without a protective ileostomy. The early detection of AL is crucial, as it can significantly impact the patient’s recovery and survival rates. This study aims to assess the diagnostic accuracy of abdominal Computed Tomography(CT) scans in identifying AL among rectal cancer patients post-LAR. This retrospective cohort study was conducted at the Tehran Surgical Research Center and included 42 patients with rectal cancer who underwent LAR after neoadjuvant chemoradiotherapy. All patients received an abdominal CT scan on the fifth postoperative day. The presence of AL was confirmed based on clinical symptoms, radiological findings, and the necessity for reoperation. The study focused on evaluating specific CT findings, such as perianastomotic collections, to determine their correlation with AL. The analysis revealed that certain CT scan findings, particularly complex collections larger than 5 cm in proximity to the anastomosis site and signs of generalized peritonitis, were significantly associated with the presence of AL. These findings were particularly important in cases where patients also exhibited clinical symptoms like fever and leukocytosis. The presence of these CT indicators often necessitated reoperation to address the leakage. The results of this study suggest that while abdominal CT scans are a valuable tool in the early detection of AL, the interpretation of these scans must be done in conjunction with clinical symptoms. The study highlights the importance of considering the size of perianastomotic collections and the presence of systemic symptoms for an accurate and timely diagnosis of AL. Future research should further investigate the potential of CT scans in improving outcomes for patients undergoing LAR for rectal cancer.

Abstract.

## Introduction

The most feared concern after colorectal surgery is serious complication of anastomotic leakage (AL). This severe complication is life-threatening if not diagnosed and treated quickly and correctly. Emergency reoperation is the first necessity. Quality of life in this condition is affected by poor functional outcomes associated with a prolonged hospital stay and high rate of permanent stoma formation^1–6^.

If the patient’s clinical condition worsens after colorectal surgery suggesting AL, and emergency laparoscopy or laparotomy should be performed to avoid potential adverse outcomes. Clinical symptoms such as leukocytosis, fever, ileus, pelvic pain, tachycardia, peritoneal reaction, delayed gastric emptying, renal failure as well as elevated C-reactive protein are highly sensitive but these are not specific for diagnosing anastomotic leakage^7^. Computed tomography (CT) is considered the preferred diagnostic modality in AL and rule out differential diagnoses. In addition to the visualization potential of AL, it has the advantage of detecting alternative complications, such as intra-abdominal abscesses. However, CT scan may lead to false negative findings due to its limited accuracy. In particular, low sensitivity leads to an increased risk of mortality as a misdiagnosis. For one optimum accuracy, knowledge of how to interpret after the operation CT findings are of great importance. The most frequent risk factors for AL mentioned are high age, male sex, a low anastomosis and malignant disease^5,6,8^.

The literature is inconclusive regarding which imaging modality is best for evaluating patients with suspected anastomotic leakage^9^. CT imaging findings may be definitive.

in anastomotic leakage, but additional imaging with aqueous solution contrast or small bowel enema may follow required to confirm leakage^10^. Standard contrast-enhanced abdominal CT scans are performed using oral and intravenous contrast. In several studies, contrast extravasation has been shown to be the only independent variable and the most reliable predictor of AL^11^. Most preferred radiologic tool for the early detection of AL is abdominal CT scan because it provides a more accurate image of the perianastomotic structures and anastomosis in comparison with standard conventional radiology. By the way, CT scan is useful in finding other postoperative complications such as intra-abdominal hematomas or abscesses mimicking the symptoms of anastomosis leakage, therefore, CT imaging is safe and can add diagnostic accuracy with high sensitivity^5,6,11–19^.

Several studies have demonstrated that contrast extravasation is the most credible and only independent factor predicting AL^5,11,20,21^.

The purpose of this study was to evaluate the value of abdominal CT scan findings in the diagnosis of anastomotic leakage and its consequences after colorectal surgery in the population of patients with rectal cancer, who underwent low anterior resection without protective ileostomy.

## Materials and methods

### Study design

This present retrospective cohort case series study was conducted using data from the Tehran Surgical Research Center, which is known for its high reliability and comprehensive coverage of rectal cancer patients in Iran. This study focused on patients who underwent surgery for rectal cancer, Low anterior resection (LAR) without protective ileostomy after 28 sessions of neoadjuvant chemoradiotherapy (CRT) and 6 weeks of rehabilitation, during 2021–2022. The pathology of the cancer was adenocarcinoma and we selected patients with lesions > 7 centimeters (cm) from anal verge in rectosigmoidoscopy. all experiments were performed in accordance with relevant guidelines and regulations. In surgical guidelines, ct scan is mentioned as one of the best modalities for detecting anastomotic leak, but it is not mentioned precisely about the findings consistent with anastomotic leak. In this study, we examined the findings that were more consistent with an anastomotic leak.

### Patient selection

Our study was conducted among 42 patients with colonoscopy-proven rectal adenocarcinoma. All patients had 28 sessions of CRT and were scheduled for surgery with a delay of 6 weeks. The operations started with a mid-line incision on the abdomen. Before starting any procedure, we had an evaluation for existing any intraabdominal and distant metastases and also tumor locally circumstances were examined and all of 42 patients were seemed operable at first look. Then Low anterior resection started with Total Mesorectal Excision (TME) plan, followed by complete mobilization of the descending colon and left flexure. After Exploring and high ligating of the inferior mesenteric artery and vein lymph node dissection and resection was performed as well. After complete Tumor resection with 5 cm margin from the tumor edges, anastomosis performed with Ethicon and Covidian Staplers and all of the anastomoses were checked for complete integrity with air insufflation under water and none of patients had any signs of primary AL. A protective ileostomy is not considered for all of these cases. Drainage were performed in all patients.

All 42 operations were performed by one experienced colorectal surgeon in our center from the beginning to the end and all patients underwent abdominopelvic CT scan (Computed Tomography scan) with intravenous contrast on the fifth day post operation. All CT scans were reported by an experienced abdominal radiologist, and the ALs were confirmed by a second radiologist independently.

**Inclusion criteria**:

1. Patients with adenocarcinoma of rectum who went under 28 session chemoradiotherapy (CRT) with 6 weeks delay for rehabilitation period and are the candidates for (LAR).
2. Stable conditions in surgeon opinion about the circumstances during surgery, including: properly preoperative mechanical bowel preparation, absence of bowel obstruction, not an emergency surgery, safe anastomosis, not a massive bleeding during surgery, normal Lab data before surgery, no other underlying diseases and no significant drug history like corticosteroids, insulin, ….
3. Tumoral lesions > 7 centimeters distance from anal verge.
4. In all patients we used standard and similar Staplers include:

- 29 Covidian annular staplers for the rectosigmoid anastomosis.
- Green Ethicon contour for rectal stump.

5. All patients had their CRT in our center with the same and safe methods and had no any significant complications within the therapy.
6. Only patients which are at the stage: T3N1.

**Excluding criteria**:

1. Patients without CRT.
2. Patients with nontreated immunocompromised disease (e.g. Corticosteroids, uncontrolled Diabetes Mellitus, Systemic Lupus Erythematous, …).
3. Patients with American Society of Anesthesiologists (ASA) score of 3 and 4.
4. Patients with any kind of complications which make surgeon to loop diverting stoma insertion like: unsafe anastomosis, massive hemorrhage during surgery, ….
5. Microscopically non-radical resections.
6. Patients with more than T3N2M0 in primary evaluation.

### Data collection (Lab data, radiological, clinical and paraclinical data)

- Complete Blood Count (CBC) diff: Daily check from the third postoperative day to 7th.
- Erythrocyte Sedimentation Rate (ESR) and C-reactive protein(CRP): Check in 4th and 5th days postoperatively.
- Abdominopelvic CT Scan with IV (Intra venous) contrast: Between days 5–7 postoperatively.
- If there were any signs of AL in CT scan, patients scheduled for rigid sigmoidoscopy and if there were not any signs of AL (Healthy anastomosis) patients dismissed from hospital on day 7 postoperatively.

In patients with positive leakage based on our practice definition, we considered reoperation including laparotomy, peritoneal irrigation and loop diversion stoma insertion. Patients were visited weekly in clinic after discharging from hospital. In patients with clinic follow up if there were any signs of AL CBC diff, CT Scan and Magnetic Resonance Imaging (MRI) were reconsidered and if AL approved, they had to be planed for delayed stoma insertion.

We considered CT Scan due to lack of statistical superiority in available sources and also because of the mortality rate of missed patients with AL.

**AL definitions**:

A. **Radiological leakage: (All of radiological examinations were reported by one individual expert Radiologist)**

1. Any abscess formation near to anastomosis.
2. Air existing out of luminal bowel.

B. **Clinical leakage**:

1. > 38 degree centigrade fever and any symptoms of peritoneal irritation.
2. Leukocytosis for more than 72 h.
3. Fecal or pus secretion from abdominal drain.
4. Peritoneal pain and pus secretion from anus.
5. CRP > 135 on day 5 postoperatively.

AL was considered as bellow finally:

A. Unexplained clinical symptoms of leakage or other complications were checked with rectosigmoidoscopy if there were any suspicion remained.
B. Radiological symptoms of leakage which confirmed by 2 Radiologists and accompanied with at least one clinical criteria and checked by rectosigmoidoscopy if there were any suspicion remained.

### Statistical analysis

#### Sample size and power calculation

A post hoc power analysis was conducted to assess whether the sample size of 42 patients was sufficient to detect a statistically significant association between CT scan findings and the presence of anastomotic leakage (AL). Assuming a medium effect size (Cohen’s w = 0.3), a significance level of α = 0.05, and using a chi-square test for proportions, the study achieved a statistical power (1 - β) of approximately 80%. This indicates that the current sample size is adequate to draw meaningful and robust conclusions regarding the diagnostic value of CT scan findings in this patient population. The effect size and parameters were based on prior similar studies and clinical relevance in colorectal surgery literature.

The obtained information was analyzed using descriptive and analytical statistics and using SPSS software version 22. Correlation relationship between patients’ data, results of abdominopelvic CT scan with intravenous contrast and reoperation was analyzed with Phi Kramer statistical test and also Shapiro-Vick test (checking the normality of data distribution) and the effects and significance level between them were investigated. P value less than 0.05 was considered significant.

## Results

42 patients in our study with rectal adenocarcinoma were prepared for Low Anterior Resection. The average age was 63.1 years, 26 (61.9%) were female and 16 (38.1%) were male. In this statistical population, 6 patients had reoperation due to AL (14%). Outcome results of the investigation carried out in this study to find the existence of a significant relationship between CT scan findings, which include opacity around the anastomosis, collection next to the anastomosis, complex collection or collection above 5 cm and generalized peritonitis, in the diagnosis of anastomotic leakage (AL) and re-operation in rectal cancer patients who underwent low anterior resection without protective ileostomy is as follows. [Tables 1 and 2]

According to Phi Cramer’s test, the correlation between CT scan findings listed below and AL is confirmed.

**Table 1:** Correlation of CT scan findings with signs of anastomotic leakage (AL).

CT Scan Findings	Anastomotic leakage (AL)	*P* value
presence of collection near anastomosis	+	0.029
presence of complex collection or collection above 5 cm	+	0.000
presence of generalized peritonitis	+	0.000
positive rectosigmoidoscopy	+	0.000
presence of opacity around the anastomosis + positive rectosigmoidoscopy	+	0.000
presence of collection near the anastomosis + positive rectosigmoidoscopy	+	0.000
presence of complex collection or collection above 5 cm + fever	+	0.007
presence of complex collection or collection above 5 cm + leukocytosis	+	0.002
presence of complex collection or collection above 5 cm + positive rectosigmoidoscopy	+	0.000
presence of generalized peritonitis + fever	+	0.013
presence of generalized peritonitis + leukocytosis	+	0.013
presence of generalized peritonitis + positive rectosigmoidoscopy	+	0.000

According to Phi Cramer’s test, the correlation between CT scan findings listed below and AL is not confirmed.

**Table 2:** Negative correlation of CT scan findings with anastomotic leakage (AL).

Negative Correlation of CT Scan Findings	Anastomotic leakage (AL)	*P* value
presence of opacity around the anastomosis		0.139
presence of fever		0.900
presence of leukocytosis		0.900
presence of opacity around the anastomosis + fever		0.608
presence of opacity around the anastomosis + leukocytosis		0.800
presence of collection near the anastomosis + fever		0.209
presence of collection next to anastomosis + leukocytosis		0.276

## Discussion

There are few studies that have investigated the parameters involved in the diagnosis of anastomotic leakage by CT scan, and none of the variables and findings included in our study were evaluated in detail before. Therefore, in this study we present a new investigation on the potential relationship between CT findings and the presence of AL based on qualified evidence. In 2015, Kauv P et al. showed the most sensitive (reviewers, 83% and 83%) and specific (97% and 97%) sign was contrast extravasation which was strongly associated with Anastomotic leakage by univariate analysis (*P* < 0.0001 and *P* < 0.0001). In 2015, Huiberts et al. demonstrated that leakage of contrast medium is the only independent variable predicting Anastomotic leakage. To improve the accuracy of CT imaging, administration of optimal contrast near the anastomosis seems essential. In 2018, Samji et al. presented when an intraluminal contrast agent was used, diagnostic performance of CT was highest. Intraabdominal free fluid was the most sensitive imaging predictor (95.3%). A highly specific imaging predictor was leakage of intraluminal contrast agent (96.6%). In 2004, Eckmann et al. showed that Common clinical signs associated with anastomotic leakage were pelvic pain and fever. None of patients progressed a peritonitis. CT was the most accurate diagnostic modality (96.7%). In 2007, Power et al. emphasized that peri-anastomotic loculated fluid containing air is only characteristic seen statistically more repeatedly with CIAL. In F Mulita’s study, one of the symptoms of anastomotic leak in colorectal surgeries, surgical site infection, is mentioned. This was seen in 4 of our 6 patients who underwent reoperation and follows the findings of F Mulita’s study^22^. In another study of F Mulita Butyrylcholinesterase low levels in the first and third postsurgery were associated with an increased risk for the development of SSIs but not sepsis, which can use along with radiological facilities such as CT scan^23^.

In a meta-analysis, the accuracy of enema contrast in diagnosing anastomotic leak has been investigated. This study was conducted in 2015 and it can be said that it was the most comprehensive study regarding enema contrast. which considered it a very accurate diagnostic modality for anastomotic leak after LAR surgery. During the 10 years after this study, computerized Tomography(CT) scan systems have made tremendous progress, with the help of which the risk of false positives is much less^24^. However, if there is any diagnostic doubt in suspected and borderline patients who do not have clinical symptoms of sepsis or who are in the early stages of the disease, contrast enema and even MRI can be used. No study has yet investigated the accuracy of MRI in the early diagnosis of leak.

If there is any Complex collections or more than 5 cm collection around the anastomosis it can be said with certainty that an anastomotic leak has occurred. However, the clinical symptoms and examination of the patient should be taken into account if these symptoms are present in the CT scan. Fever, leukocytosis and positive rectosigmoidoscopy must be consider seriously and they mean that you have to take an action as soon as possible. It seems that an early CT scan doesn’t have a significant role in preventing laparotomy because the only treatment for the anastomotic leak is washing and irrigation and performing an ileostomy diversion to prevent the leak from continuing, but it can be definitely said that an early CT scan can prevent extensive sepsis and mortality in all patients, especially those who did not have a prophylactic loop ileostomy in their first operation.

According to the limited studies conducted in this field, it is strongly recommended that patients with rectal cancer who are candidates for low anterior resection (LAR) without prophylactic ileostomy with close observation CT scans should undergo surgery in well-equipped centers with the presence of experienced radiologists.

Future studies with larger sample sizes and multicenter validation is suggested to confirm the findings.

It is also recommended that future studies consider the size of the defect observed at the anastomosis site during rectosigmoidoscopy as a factor in determining the treatment plan.

## Conclusion

AL is a horrible event in patients with intraabdominal intestinal anastomoses. In the hand of best surgeons in the best quality and facility centers the rate of AL is inevitably 8–10%. We can prevent from mortality by early AL diagnosis and a proper modality is CT scan plus clinical manifestations and we used rectosigmoidoscopy as well. The presence of opacity and even collection around the anastomosis alone is not a reliable factor to decide about AL. The most important factor is the size of collection. If there were any complex collections or more than 5 cm collection around the anastomosis it can be said with certainly that AL is positive. The presence of fever, leukocytosis and positive rectosigmoidoscopy will strengthen the diagnosis and we can perform a treatment as soon as possible.

## Data Availability

The datasets generated and/or analyzed during the current study are available from the corresponding author on reasonable requestAll materials used in this study are available from the corresponding author on reasonable request.

## Declarations

the treatment process to writing the manuscript.
